# Longitudinal Changes in Motor Estimation Error and Motor Function in Patients with Parkinson’s Disease: A Case Report

**DOI:** 10.3390/medicines10070042

**Published:** 2023-07-06

**Authors:** Katsuya Sakai, Tsubasa Kawasaki, Hiroya Kiminarita, Yumi Ikeda

**Affiliations:** 1 Department of Physical Therapy, Faculty of Health Sciences, Tokyo Metropolitan University, Tokyo 116-8551, Japan; k.sakai@tmu.ac.jp (K.S.); ikedayum@tmu.ac.jp (Y.I.); 2 Department of Physical Therapy, School of Health Sciences, Tokyo International University, Kawagoe 350-1197, Japan; 3 Department of Rehabilitation, Kirameki Visiting Nursing Rehabilistation, Kawagoe 350-0033, Japan; kimi.hatch.08@gmail.com

**Keywords:** Parkinson’s disease, motor estimation error, longitudinal study, case reports, motor imagery

## Abstract

Background and Objectives: This report described two cases with clear longitudinal changes in motor estimation error (difference between the motor imagery and motor execution) and their progression and motor and activities of daily living (ADL) function changes in patients with PD. Materials and Methods: Patient 1 was a 68-year-old man (Hoehn and Yahr [H and Y] stage: IV, diagnosed with PD for 11.8 years) and patient 2 was a 68-year-old woman (H and Y stage: II, diagnosed with PD for 9.6 years). Imagined two-step test (iTST), two-step test (TST), and PD-related assessments (Unified Parkinson’s Disease Rating Scale [UPDRS], and Freezing of Gait Questionnaire [FOGQ]) were assessed at baseline and after 6 months. Motor estimation error was calculated as the iTST distance minus TST distance. Results: In patient 1, motor estimation error was greater after 6 months (baseline: 5.7 [4.8%]/after 6 months: 25.7 cm [26.1%]). Moreover, UPDRS and FOGQ total scores deteriorated after 6 months (UPDRS total: 29/34 point, and FOGQ: 9/16 point). Conversely, in patient 2, motor estimation error did not change notably (−3.6 [7.6%]/−2.5 cm [7.0%]), while UPDRS and FOGQ total scores improved after 6 months (UPDRS total: 17/12 point, and FOGQ: 6/1 point). Conclusions: This report indicated that greater motor estimation error may be associated with declining motor and ADL function and disease progression in patients with PD.

## 1. Introduction

Motor imagery (MI) is the mental planning of motor execution and is reported to elicit brain activity similar to that in motor execution [1,2,3]. MI ability shows a decline in older people and in patients with certain diseases, including stroke and Parkinson’s disease (PD) [4,5,6]. MI ability more frequently declined in patients with PD than that in older people [4,7]. In addition, it was associated with lower motor function [4]. The brain activity of patients with PD during MI was also lower than that of older people, resulting in lower motor function [7,8]. Additionally, the difference between MI and motor execution, which is known as motor estimation error, may influence motor function [4,8,9,10,11]. Motor estimation error indicates the ability to accurately recognize one’s body movements [4,8,9,10,11]. Motor estimation error is evaluated in the following manner. When MI is greater than the actual motor execution, participants are considered to overestimate their physical function [11]. When MI is less than the actual motor execution, participants are considered to underestimate their physical function [11].

Sakurai et al. [8] investigated motor estimation error by calculating the difference between the imagined height at which a bar could be stepped over and the actual stepped height in three age groups: young adults, young-old adults (age 60–74 years), and old-old adults (age > 74 years). The results showed that the imagined step-over heights of the three groups were not significantly different; however, the actual step-over height significantly decreased with increasing age (actual step-over height: young > young-old > old-old). Furthermore, a 3-year follow-up by Sakurai et al. [12] showed that the overestimation of physical function in older people increased from 10.3% to 22.4%, which was accompanied by a significant decline in physical function (i.e., walking speed and step-over height) in comparison to that at baseline. Moreover, Kawasaki et al. [11] investigated the relation between two-step distance, walking speed, and motor estimation error in older people. The results showed that older people who overestimated their physical function had shorter two-step distance and slower walking speed. Therefore, in older people who overestimate their performance, the actual motor function is lower.

Motor estimation error is related to motor function in patients with PD, as well as in older people [4,13,14]. Kawasaki et al. [13] investigated motor estimation error using a two-step test (TST) in patients with PD and healthy older people. The results demonstrated that the overestimation of physical function was greater in patients with PD than in healthy older people. Additionally, the patients with PD who overestimated their motor function had significantly higher scores on the Unified Parkinson’s Disease Rating Scale (UPDRS) part Ⅱ and Ⅲ (ability of activities of daily living [ADL] and motor function were lower). In another study of persons with PD investigating the correlation between motor estimation error (based on the TST) and motor function, motor estimation error showed significant correlations with UPDRS part II and Hoehn and Yahr (H and Y) stage [14].

As mentioned earlier, the motor estimation error was overestimated in older people due to their declined motor function in a 3-year follow-up longitudinal study [12]. However, whether patients with PD had similar longitudinal changes in motor estimation error as the older people and what their progress and terms were remain unclear. PD is a progressive disease and should cause a decrease in motor function faster than the rate of decline in older people. This case report describes two cases with clear longitudinal changes in motor estimation error and their progression. It also reports the motor function changes based on detailed clinical data. It was found that, in patient 1, longitudinal change in the motor estimation error shifted to overestimation, and motor and ADL function declined compared to baseline after only 6 months. However, though patient 2 also exhibited a longitudinal change, the motor estimation error did not change notably. The motor and ADL function were maintained after 6 months compared to the baseline. In the future, the motor estimation error may be used as an indicator to identify disease progression and declining motor and ADL function. Therefore, this case report aimed to highlight the relationship between clear longitudinal changes in motor estimation error (based on the TST) and their progression and motor and ADL function in two patients with PD.

## 2. Materials and Methods

Two outpatients with PD participated in this study (Table 1). Both patients could step and maintain a standing position without any support and had no visual or cognitive impairment. Patient 1 was a 68-year-old man who was diagnosed with PD for 11.8 years. He presented with tremors in his right upper and lower extremities and a short-stepped gait. The L-dopa dose at the onset was 300 mg/day. He also reported progressive tremors, immobility, postural dysreflexia, freezing of gait, and on–off phenomenon for over 9 years. At the start of this case report, he was administered L-dopa (total 900 mg/day, 4 times a day, every 3 h), and was diagnosed with H and Y stage IV. He could walk around his house holding onto the handrail or walking aids; however, he had difficulty going outside. He had undergone home-rehabilitation therapy for 120 min per week (one time 60 min) for the past 2 years, focusing primarily on stretching (average 10 min), balance (average 20 min), and walking training (average 30 min). In particular, walking training was performed in and around the home. Patient 2 was a 68-year-old woman who was diagnosed with PD for 9.6 years. She presented trunk flexion posture and gait disturbance but could walk indoors and do housework. However, progressive tremors, postural dysreflexia, and gait disturbance have been reported for over 7 years. Therefore, she was diagnosed with H and Y stage II, and going outdoors was difficult at the start of this case report. She was not administered L-dopa and did not report an on–off phenomenon. Therefore, she had undergone home-rehabilitation therapy for 60 min per week for the past 2 years, mainly for balance and walking training. Balance training consisted mainly of stepping exercises and one-legged standing while holding the trunk in extension. Walking training were performed in and around the home while increasing the stride length. These assessments and rehabilitation were performed at the same time and by the same person. The purpose of this study was explained to both patients, and written informed consent was obtained from them. This study was conducted with the approval of the institutional ethics committee of the Tokyo Metropolitan University Arakawa Campus (approval number: 19026) and complied with the ethical standards laid down in the 1964 Declaration of Helsinki.

**Figure 1:**
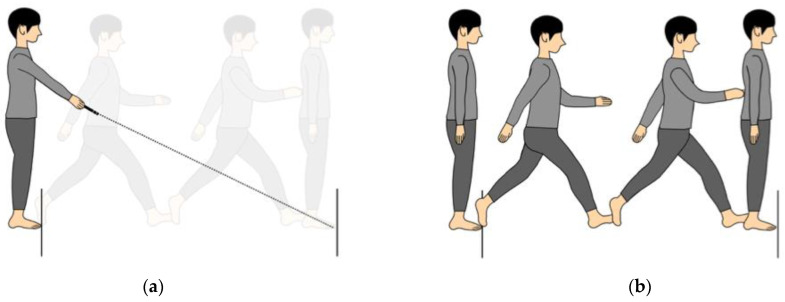
(**a**) Illustration of the imagined two-step test. In this test, participants used a laser pointer to indicate their imagined maximum two-step distance. (**b**) Illustration of the actual two-step test. The participants first performed the imagined two-step test and then completed the maximum two-step.

The assessments were measured at baseline and after 6 months. The assessment items consisted of TST [13,14,15], imagined two-step test (iTST) [13,14], PD-related assessments, and motor estimation error-related questionnaires. PD-related assessments included UPDRS [16], and Freezing of Gait Questionnaire (FOGQ) [17], while the motor estimation error-related questionnaires were the Fall Efficacy Scale (FES) [18], Hospital Anxiety and Depression Scale (HADS) [19], and presence or absence of falls. First, iTST was conducted, followed by TST (Figure 1a,b) [13,14]. The patients performed the iTST using a laser pointer to indicate the imagined maximum two-step distance (Figure 1a), which the physical therapist then recorded. The iTST distance was only measured once after the physical therapist explained the method and confirmed that the patient understood it. This is because the imagined distance is influenced by the repetition of the test [11,13,14]. After performing the iTST, the patients took a maximum of two steps forward from the line of a measure delineated by the physical therapist to measure the TST distance. The TST distance was then recorded. (Figure 1b). The TST had been validated in older people [15]. The TST distance was measured from toe to toe, and maximum two-step distances were measured by the physical therapist. The maximum TST distance was measured only once as conducted in iTST. The motor estimation error distance was calculated using the following formula: iTST distance minus TST distance [11,13,14]. When the motor estimation error is from −5 cm to +5 cm, over +5 cm, and under −5 cm, the patient is regarded as having appropriate estimation, overestimation, and underestimation of his or her physical function, respectively [11]. In addition, to standardize the motor estimation error, the following formula was used: (TST − iTST)/TST × 100 [5].

The UPDRS consists of four parts [16]: part I assesses mental function, behavior, and mood; part II, ADL; part III, motor function; and part IV, treatment complications. The total UPDRS score is 199 points, with part I, II, III, and IV consisting of 16, 52, 108, and 23 points, respectively. Higher UPDRS scores indicate increased PD severity.

We also assessed the freezing of gait, fall-related self-efficacy, and anxiety and depression. The FOGQ is a six-item questionnaire that assesses the degree of subjective freezing of gait [17]. Each item is rated on a scale of 0–4 points, with a total score of 24 points. Higher FOGQ scores correspond to increased degree of gait freezing.

The FES assesses a respondent’s fear of falling in daily life [18]. FES had been validated in patients with PD [19]. It consists of 10 items rated on a scale of 0–4 points, with a total score of 40 points. Lower FES scores relate to a greater fear of falling.

The HADS assesses anxiety and depression, consisting of 14 items [20]. HADS had been validated in patients with PD [21]. Each item is rated on a 4-point scale from 0–4 points, with higher scores associated with higher anxiety and depression levels.

## 3. Results

The results of all assessment items at baseline and after 6 months are shown in Table 2. In patient 1, the TST distance after 6 months was shorter than that at baseline. However, the iTST distance was similar at baseline and after 6 months. Therefore, the motor estimation error distance indicated overestimation after 6 months (baseline: 5.7 cm [4.8%], and after 6 months: 25.7 cm [26.1%]). Moreover, UPDRS part II–IV and FOGQ scores deteriorated from baseline to after 6 months. The worsened items of UPDRS were turning, upper limb movement, and oral function. However, the H and Y stage IV and L-dopa dose did not change from baseline to after 6 months. Finally, FES and HADS anxiety scores deteriorated from baseline to after 6 months. He had difficulty going out because of the increased freezing of gait at gait initiation and change in direction.

In patient 2, the motor estimation error distance did not show any notable changes after 6 months (baseline: −3.6 cm [7.6%], and after 6 months: −2.5 cm [7.0%]). Additionally, the PD-related assessments of patient 2 and UPDRS part I–III, and FOGQ scores improved from baseline to after 6 months. The improved items of UPDRS were motivation, walking, facial expression, and finger tapping. However, the H and Y stage did not change from baseline to after 6 months. Lastly, FES and HADS depression scores improved from baseline to after 6 months. She could walk outdoors.

## 4. Discussion

This study reported two cases with clear longitudinal changes in motor estimation error and motor and ADL function changes as PD progresses. The motor estimation error of patient 1 indicated overestimation after 6 months when compared to the baseline. Additionally, UPDRS part II–Ⅳ, FOGQ, FES, and HADS anxiety scores deteriorated from baseline to after 6 months. Conversely, though the TST and iTST distances of patient 2 equally declined from baseline to after 6 months, the motor estimation error did not change notably after 6 months. UPDRS part I–III, FOGQ, FES, and HADS depression scores improved from baseline to after 6 months. Therefore, patient 1 who overestimated his physical function showed a decline in motor and ADL function. However, patient 2 who appropriately estimated her physical function maintained motor and ADL function. This report indicated that overestimation based on motor estimation error may be associated with declining motor and ADL function in patients with PD. However, two cases were in different stages, disease progression, and had different levels of motor impairment. Therefore, these must be taken into consideration.

Motor estimation error was associated with TST, UPDRS, and FOGQ. In Patient 1, the TST distance after 6 months was shorter than that at baseline. However, the iTST distance was similar value at baseline and after 6 months. Therefore, the motor estimation error distance indicated overestimation after 6 months. The reason for this change, we speculated to be related to a decline in physical function based on the results of previous studies [13,14]. A previous cross-sectional study reported measured motor estimation error using TST in a patient with PD and investigated the relationship between the motor estimation error and UPDRS part II–III score (ADL and motor function) [13]. The result showed that motor estimation error significantly correlated with the UPDRS part II–III score (i.e., overestimation indicated lower ADL function, and physical function). Another previous study reported motor estimation error using TST in PD patients and the relationship between the motor estimation error and H and Y stage [14], indicating that motor estimation error significantly correlated with H and Y stage. The severity of patients with PD, UPDRS, and degree of motor estimation error in this previous study was similar to that of the patients in the current study. Therefore, the current case report may support the findings of cross-sectional studies [13,14]. In addition, motor estimation errors in patients with PD showed similar changes to those in older people and patients with stroke [12,22]. Moreover, UPDRS part II was 3 points, part III–IV was 1 point each, and FOGQ was 7 points worse. FOGQ changed beyond Minimal Detectable Change (MDC) [23]. Patient 1 had worsened motor and ADL function as well as FOG. He had difficulty going outside his house because of the increased freezing of gait at gait initiation and change in direction. The TST and iTST distances of patient 2 equally declined from baseline to after 6 months, but the motor estimation error did not change notably after 6 months. However, UPDRS parts II and III both decreased by 2 points, and FOGQ by 5 points. Therefore, motor estimation error did not change notably and improved scores of PD symptom. In addition, FOGQ did change beyond MDC [23] and she could walk outdoors. Therefore, we speculated that motor estimation error was overestimated due to the decrease in TST distance and associated with disease progression.

In addition, motor estimation errors in patients with PD may change rapidly. Sakurai et al. [12] reported that motor estimation errors in older people change longitudinally when overestimating the influence of motor function decline, inactive lifestyle, falls, and frequency of outings in a 3-year follow-up longitudinal study. They also investigated motor estimation error by calculating the difference between the imagined height at which a bar could be stepped over and the actual stepped height in older people at baseline and after 3 years. Their results indicated that motor estimation error shifted to overestimation after 3 years. In the current study, the motor estimation error of patient 1 shifted to overestimation after only 6 months. Therefore, motor estimation errors in patients with PD may change more rapidly than in older people. However, it is imperative to note that this study was not compared with older people and involved only two patients. In addition, changes in motor estimation error need to be associated with various outcomes, such as motor function and severity of the disease. However, we considered it necessary to present this report discussing the rapid changes in motor estimation errors in patients with PD.

Overestimation could also be due to the reduced frequency of going out associated with an inactive lifestyle in older people [12]. A lower frequency of going out leads to inactivity, which in turn reduces the opportunity to recognize one’s physical function. Those individuals might not have access to any recent experiences to apprise their own physical function, which might lead them to overestimate their physical function. The frequency of going out in the current study was assessed using the Life-Space Assessment (LSA) [24]. The LSA score of patient 1 was lower than that of patient 2. Therefore, patient 1 could have overestimated his physical function due to the absence of recent experiences required for recognizing the physical function changes that were caused by the decline in motor function and disease progression. Conversely, in patient 2, the motor estimation error distance could have been maintained by the utilization of recent experiences for recognizing changes in the physical function as motor function improved and she could walk outdoors.

Moreover, the difference in MI ability may affect motor estimation error. MI ability is higher in people who exercise than those who do not exercise [25,26]. Furthermore, a previous study reported that inactive older people have lower MI ability and difficulty with imagery accuracy [12]. Therefore, patient 1 showed an increase in motor estimation error due to overestimation of physical function while patient 2 maintained motor estimation error.

Additionally, both patients did not report occurrence of falling at baseline and after 6 months. The FES score of patient 1 who overestimated his physical function exhibited a deterioration in the FES score, indicating an increase in fear of falling from baseline to after 6 months. However, patient 2 improved from baseline to after 6 months. This is in line with studies that reported an association between overestimation of physical function and falls and fear of falling [5,9,10,12]. Therefore, maintaining motor function may prevent a shift to the overestimation of physical function and may decrease falls and fear of falling.

Focus should also be laid on the changes in HADS scores. Patient 2’s HADS anxiety score after 6 months had a low score and depression score improved than baseline. This may have been influenced by improvements in motor and ADL function, and expansion of the life space. It was reported that increased physical activity and improved motor function are associated with decreased anxiety and depression in patients with PD [27,28].

This study has some strengths and limitations. This study is the first to reveal that motor estimation errors longitudinally change to overestimation as motor and ADL function declines in patients with PD. Thus, motor estimation error has shown the potential to be used as an assessment to detect motor function decline and fall in PD as well as in the elderly people. However, this study included only two participants who were not in the general disease progression process and had different degrees of severity. Therefore, it is difficult to generalize the results. Finally, cognitive function was measured using MMSE only. Therefore, a comprehensive assessment of cognitive function is needed. However, we thought it important to report the motor estimation error and the rapid changes in motor function in patients with PD. We will address the limitation of a small sample by increasing the number of participants. In addition, the amount of activity performed by the patients was not measured. We will address this by measuring the amount of physical activity in future studies. In the future, the motor estimation error may be used as an indicator to capture disease progression and decline in motor and ADL function.

## 5. Conclusions

Greater motor estimation error may be associated with motor and ADL function decline and disease progression in patients with PD. However, this study included only two cases with different stages, disease progression, and different levels of motor impairment. Therefore, future study will need to analyze more cases in order to generalize the results.

## Figures and Tables

**Table 1:** Demographic characteristics of the patients.

	Patient 1	Patient 2
Age (years)	68	68
Male/Female	Male	Female
Height (cm)	155	153
Weight (kg)	53	44.8
BMI (kg/m^2^)	22.06	19.14
MMSE (score)	29	30
LSA (score)	31	58
Rehabilitation time (min/week)	120	60
Hoehn and Yahr	Ⅳ	Ⅱ
L-dopa dose (mg/day)	900	0

BMI: body mass index, MMSE: mini-mental state examination, LSA: life-space assessment.

**Table 2:** Assessment of the patients.

	Patient 1		Patient 2	
	Baseline	After 6 Months	Baseline	After 6 Months
TST (cm)	118.9	98.5	124.2	114.7
iTST (cm)	124.6	124.2	120.6	112.2
Motor estimation error distance (cm)	5.7	25.7	−3.6	−2.5
Motor estimation error distance (%)	4.8	26.1	7.6	7.0
Hoehn and Yahr	IV	IV	II	II
UPDRS part I (score)	1	1	1	0
UPDRS part II (score)	13	16	6	4
UPDRS part III (score)	11	12	10	8
UPDRS part IV (score)	4	5	0	0
UPDRS total (score)	29	34	17	12
FOGQ (score)	9	16	6	1
L-dopa dose (mg/day)	900	900	0	0
FES (score)	29	27	21	27
HADS anxiety (score)	4	6	1	1
HADS depression (score)	3	3	18	11
MMSE (score)	29	29	30	26
LSA (score)	31	32	58	60
Occurrence of falls	Absence	Absence	Absence	Absence

TST: two-step test, iTST: imagined two-step test, UPDRS: unified Parkinson’s disease rating scale, FOGQ: freezing of gait questionnaire, FES: fall efficacy scale, HADS: hospital anxiety and depression scale, MMSE: mini-mental state examination, LSA: life-space assessment.

## Data Availability

Data are available from the corresponding author upon request.
